# Misleading Advertising for Antidepressants in Sweden: A Failure of Pharmaceutical Industry Self-Regulation

**DOI:** 10.1371/journal.pone.0062609

**Published:** 2013-05-01

**Authors:** Anna V. Zetterqvist, Shai Mulinari

**Affiliations:** 1 Department of Clinical Sciences, Lund University, Malmö, Sweden; 2 Department of Gender Studies, Lund University, Lund, Sweden; University of British Columbia, Canada

## Abstract

**Background:**

The alleged efficacy of pharmaceutical industry self-regulation has been used to repudiate increased government oversight over promotional activity. European politicians and industry have cited Sweden as an excellent example of self-regulation based on an ethical code. This paper considers antidepressant advertising in Sweden to uncover the strengths and weaknesses of self-regulation.

**Methodology:**

We analyzed all antidepressant advertisements in the Swedish Medical Journal, 1994–2003. The regulation of these advertisements was analyzed using case reports from self-regulatory bodies. The authors independently reviewed this material to investigate: (1) extent of violative advertising; (2) pattern of code breaches; (3) rate at which the system reacted to violative advertising; (4) prevalence of and oversight over claims regarding antidepressant efficacy and disease causality, and (5) costs for manufactures associated with violative advertising.

**Principal Findings:**

Self-regulatory bodies identified numerous code breaches. Nonetheless, they failed to protect doctors from unreliable information on antidepressants, since as many as 247 of 722 (34%) advertisements breached the industry code. Self-regulatory bodies repeatedly failed to challenge inflated claims of antidepressant efficacy, lending evidence of lax oversight. On average, 15 weeks elapsed between printing and censure of a wrongful claim, and in 25% of cases 47 weeks or more elapsed. Industry paid roughly €108000 in fines for violative advertising, adding an estimated additional average cost of 11% to each purchased violative advertisement, or amounting to as little as 0.009% of total antidepressant sales of around €1.2 billion.

**Conclusions:**

Lax oversight, combined with lags in the system and low fines for violations, may explain the Swedish system’s failure to pressure companies into providing reliable antidepressants information. If these shortcomings prove to be consistent across self-regulatory settings, and if appropriate measures are not taken to amend shortcomings, many countries may want to reconsider the current balance between self-regulation, and legislative control with government oversight.

## Introduction

Pharmaceutical companies advertise in medical journals to exert influence over doctors’ prescribing habits [1]. Industry representatives assert that advertisements serve to educate doctors and support rational prescribing practices [2]–[3]. However, a growing number of international studies investigating the quality of claims made in advertisements have reached the conclusion that claims, especially in developing countries, are often incomplete, inflated and sometimes downright misleading [4]–[14], which could result in inappropriate prescribing practices [15]. For example, a study investigating the accuracy of psychiatric medication advertisements in high-impact medical journals in the United States found that most claims in advertisements provided either no attainable source (50%) or, when sources could be attained, contained references that failed to support the claim (45%), leading the authors to recommend increased regulation of such advertising [11].

Given this research evidence pointing to the low quality of medical journal drug advertisements as a global problem that could endanger public health, the time is ripe to scrutinize the promotion regulatory mechanisms that are in place to ensure truthful and meaningful information on drugs [2], [16]–[19]. To this end, this paper considers the workings of the Swedish regulatory system, which has previously been singled out as particularly efficient and trustworthy [16]. The importance of considering this regulatory system was underscored more recently when European politicians and industry representatives cited Sweden, in the context of the new European Union patient information proposal, as an excellent example of how industry ensured supposedly reliable information on drugs over an extended period of time [20]. Thus, the European Federation of Pharmaceutical Industry and Associations (EFPIA) pointed to the positive experience with industry-based medicines information in Sweden and the United Kingdom to argue that “self-regulation by the pharmaceutical industry has proven to be highly efficient and valuable [21].” Similarly, the Pharmaceutical Forum, a high-level political platform for European discussions on pharmaco-regulatory topics, used the Swedish example, among other, to support their recommendations that “self-regulatory mechanisms should be setup within the public-private-partnership or collaborations” to ensure reliable information to patients on diseases and treatment options [22].

In Sweden, as in various other countries, including Australia, Canada, Estonia, Italy, Netherlands, Norway, Uganda, United Kingdom, Venezuela and Zimbabwe, promotional activity targeting health professionals is governed by a voluntary code of practice administered by the pharmaceutical industry’s own system of self-regulation [16], [18], [23]–[25]. The Swedish industry code was originally adopted in 1969 by representatives of the national and international pharmaceutical industry, but has been repeatedly revised [26]. The present code is essentially a modified version of what was adopted by the EFPIA [27] and the International Federation of Pharmaceutical Manufacturers Associations (IFPMA) [28]. The section of the code relating to printed promotional information targeting healthcare personnel comprises 20 articles [26]. These articles set out rules to ensure that printed information includes “accurate, objective, meaningful and balanced particulars dealing adequately with the favourable and unfavourable properties of the drugs” (article 1). More specifically, the code commands, for instance, that drug information should be within the formulation of the Summary of Product Characteristics (SPC) approved by the Swedish Medical Products Agency (MPA) (article 2) and that information “must be truthful and may not contain any presentation in words or pictures that directly or indirectly – by implication, omission, distortion, exaggeration or ambiguity – is intended to mislead” (article 4). Regarding comparative claims, the code specifies that a study that is contradicted by another/other studies may not be referred to without reservation (article 11) and that “the facts which the comparison is intended to clarify and the limitations inherent in the comparison must be stated in such a way that the comparison is not likely to mislead” (article 12).

**Figure 1:**
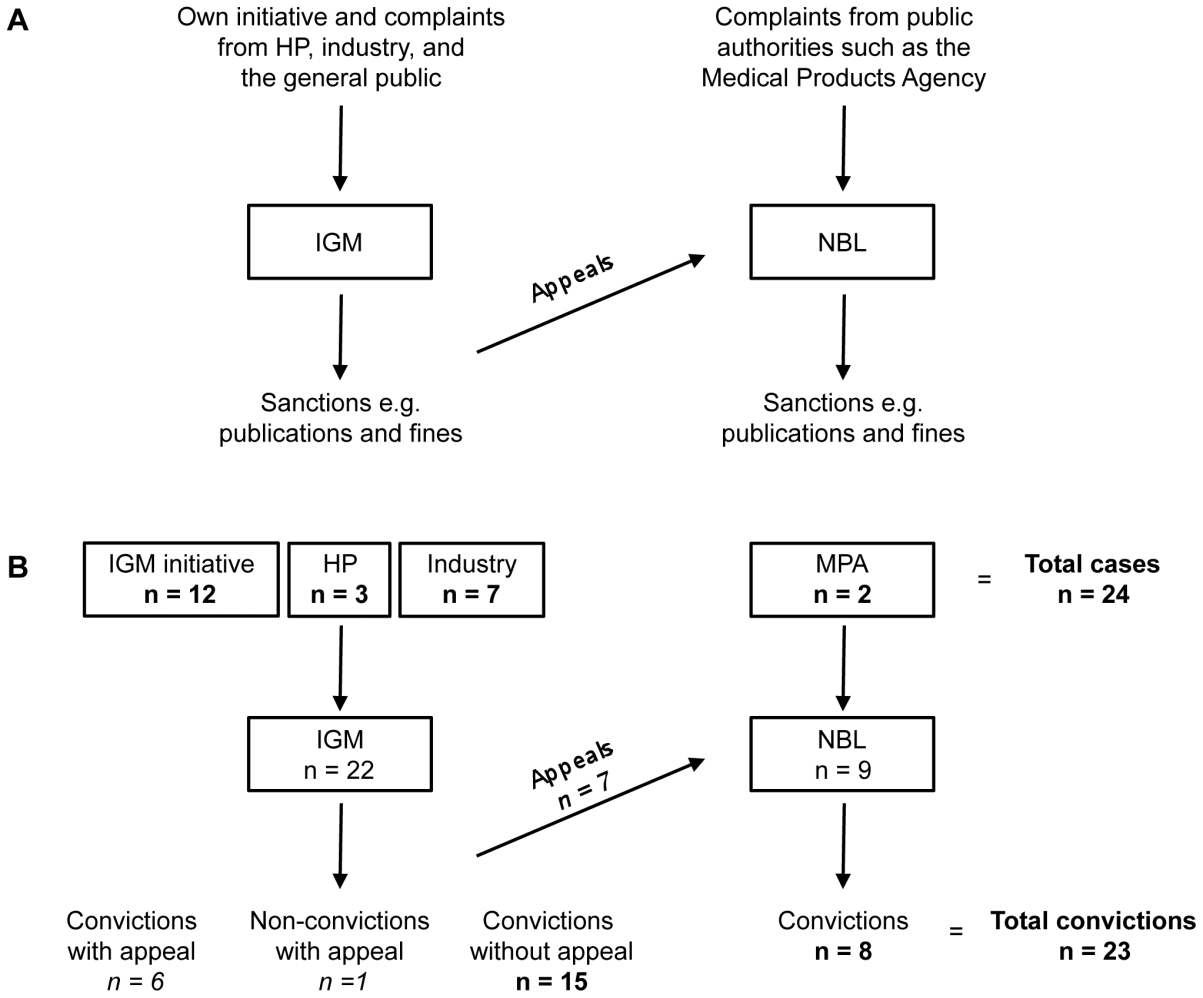
Flow through the Swedish complaints system for medicines promotion. (A) Outline of the Swedish self-regulatory system. The Pharmaceutical Industry’s Information Examiner (IGM) monitors industry promotional material for potential code breaches. The IGM also handles complaints from health professionals (HP), pharmaceutical companies and the general public. Offending companies are sanctioned by the IGM. Besides handling appeals of IGM decisions, the Information Practices Committee (NBL) considers complaints from public authorities, such as the Medical Products Agency (MPA), and sanctions code breaches. (B) Overview of the flow of cases concerning antidepressant advertising in the Swedish Medical Journal in 1994–2003 including number of cases instigated by various actors. Of the 24 cases considered by the IGM/NBL, 23 were found to be in violation.

In addition to being aligned with international industry codes of practice, the Swedish industry code is in compliance with Swedish and European Union marketing and pharmaceutical regulations. In effect, the MPA, which is obliged by law to enforce these regulations, has delegated this responsibility to the Swedish Association of the Pharmaceutical Industry (LIF). Since 1974, two main self-regulatory bodies supervise company adherence to the code: the Pharmaceutical Industry’s Information Examiner (IGM) and the Information Practices Committee (NBL) (Fig. 1A). The IGM is a scientifically qualified physician appointed by LIF, dedicated to monitoring whether medicines information from manufacturers complies with the industry code. The IGM acts either in response to complaints filed by citizens, health professionals or corporations or on personal initiative (i.e. in the absence of complaints), but does not address complaints from public authorities such as the MPA, which are sent to the NBL committee. This committee consists of a chairperson and eleven members: six represent industry, three the general public and two are medical experts. LIF appoints the entire committee; however, the representatives of the general public and the two medical experts are appointed following consultation with an appropriate body or authority representing consumers and the Swedish Medical Association, respectively. The IGM can opt to refer issues directly to the NBL without first examining them. The NBL also addresses appeals of IGM decisions. Administrative fines finance this self-regulatory system, including costs associated with the IGM and NBL. In cases of violation, fines are paid by the sanctioned company; however if a complaint brought by one pharmaceutical company against another company is ruled invalid, the complainant company pays the fee [26].

It has been suggested that extensive use of the complaints system by the MPA, industry and health professionals is an indication that the system is generally competent and unbiased [16]. Indeed, to our knowledge the ability of the Swedish self-regulatory regime to ensure reliable information on drugs has gone uncontested. In contrast, in the United Kingdom, the 2005 House of Commons Health Select Committee’s report on the influence of the pharmaceutical industry concluded that “the examples cited to us of breaches of advertising regulations, cover-up of negative medicines information and provision of misleading information to prescribers suggest that self-regulation is not working satisfactorily [23].” Among other things, the report expressed concerns about lax oversight over medicines promotion, substantial lags in the self-regulatory system that allow firms to continue running misleading advertisements for extended periods of time, and insufficient sanctions that fail to deter companies from providing unreliable information.

Motivated by such long-standing concerns, and by the citing of Sweden as a role model for pharmaceutical industry drug information provision and oversight, we investigated how the Swedish regulatory system succeeded in ensuring reliable information on antidepressants in medical journal advertisements between 1994 and 2003. This period saw an unprecedented upsurge in the antidepressants market, coinciding with the product launch of several SSRI and SNRI-type antidepressants. Pertinent to this study’s rationale, 1994 to 2003 also comprises the period of intense advertising for antidepressants, as patents for major brands expired towards the end of the period, strongly reducing the incentive to invest in drug promotion after this [29]. Here, we present data suggesting that self-regulation in Sweden largely failed to pressure companies into providing reliable information on antidepressants in medical journal advertisements.

## Methods

### Study Overview

We used antidepressant advertisements printed in the Swedish Medical Journal to investigate the workings of the Swedish self-regulatory system of medicines promotion.

1. All antidepressant advertisements in the journal between 1994 and 2003 were collected together with basic information such as date of printing.
2. Collected advertisements were coded into types according to predefined criteria (e.g. professing product efficacy in the treatment of depression, professing monoamine theories).
3. Case reports from the IGM/NBL were reviewed to identify code breaches involving antidepressant advertising between 1994 and 2003.

Information obtained from these sources were used to:

- Calculate the number of violative antidepressant advertisements over the period.
- Investigate the pattern of article breaches found by the IGM/NBL.
- Investigate the prevalence of, and oversight on the part of the IGM/NBL over, two types of claims: exaggerated statements of efficacy and claims of disease causality related to brain monoamine disturbances.
- Study the rate at which the self-regulatory system reacted to violative antidepressant advertising.
- Investigate the economic costs for drug manufacturers associated with purchasing violative antidepressant advertisements, including the magnitude of the administrative fines in relation to the cost of buying an advertisement.

### Antidepressant Advertisements in the Swedish Medical Journal

The Swedish Medical Association has published the Swedish Medical Journal (Läkartidningen) weekly since 1904. It is the official organ of the association and the only journal in Swedish indexed in Medline. The Swedish Medical Journal’s current circulation is 40 900, and readers include 74% of the country’s around 35 000 physicians, making it Sweden’s premier medical journal [30].

We searched all journal issues dating from January 1994 through December 2003 to gather unique antidepressant advertisements, i.e. advertisements of a particular kind with respect to content [11]. Since each unique advertisement usually appears many times, we also counted the times each unique advertisement was printed. The date of printing of each advertisement was recorded for subsequent calculations of the time between date of original printing and date of ruling on a wrongful claim (see below). Moreover, for subsequent cost calculations, we coded advertisements according to the standard rate categories available to advertisers that distinguish between advertisements on the basis of color and dimensions as well as the journal page on which they appear (e.g. [31]). Data were retrieved and coded independently by the two authors. In cases of discrepancy between authors’ assessments, a consensus decision was reached following a joint retrieval and coding of the data.

### IGM/NBL Cases

To identify reported code breaches we reviewed reports of cases considered by the IGM or NBL that are publically accessible in a database managed by LIF [32]. Such reports typically include a summary of specific charges, a response from the offending company, comments from the IGM/NBL, the article(s) of the code that have been breached, imposed economic sanctions, date of ruling, last date of allowed dissemination of a claim (normally 2–3 weeks from date of ruling) and, when relevant, a scanned copy of an offending advertisement. Information is not typically provided, however, about where offending advertisements were printed. The database can be searched using the Anatomical Therapeutic Chemical (ATC) code of a drug or drug class to retrieve relevant case reports. In cases where the IGM/NBL identified breaches involving antidepressants promotion (ATC code N06A) between 1994 and 2003 we assessed whether these involved one or more advertisements in our sample. For this, we used the information on specific charges and the scanned copy of an offending advertisement enclosed in the case reports. In this way, we coded all advertisements into violative or non-violative. For violative advertisements, we also recorded the violated articles of the code as specified in the case report. Again, we retrieved and coded the data independently, and in cases of discrepancy we reached a consensus decision following a joint retrieval and coding of the data. We also reviewed the case reports for evidence of repeat violations, i.e. similar violative claims for the same drug in more the one case report.

### Prevalence of and Oversight Over Claims Regarding Antidepressant Efficacy and Disease Causality

To evaluate the strength of regulatory oversight, we considered two classes of promotional claims that have been widely criticized: (1) claims pertaining to drug efficacy in the treatment of depression, and (2) claims pertaining to the biological underpinnings of depression. With respect to claims about drug efficacy, we considered two types of claims: *general claims* about antidepressant efficacy that did not make direct product comparisons, and *comparative claims* that did. All advertisements containing such claims were identified and compared to retrieved case reports to identify breaches uncovered by the IGM/NBL related to these claims.

According to the Swedish MPA, the percentage of responders (defined as >50% reduction of the baseline Hamilton Depression Scale score) in submitted studies for product approval of antidepressants varied between 13.6% and 67.9% (average 48%) for active drugs, while the corresponding rate for placebo was between 7.5% and 55.4% (average 32%) [33]. Claims of response rates of 70% or more are thus inconsistent with the submitted data on which product approval was based. Nor are such claims consistent with similar meta-analyses [34]–[36]. For this reason, we considered claims of response rates of 70% or more to be misleading. We assessed all advertisements for such claims and investigated the IGM/NBL case reports to assess whether the IGM/NBL treated them as code violations. We did not assess whether advertisements mentioned the magnitude of the placebo response because reporting of placebo data is not required under LIF’s current interpretation of the code [37].

As underscored by numerous scholars, professing monoamine theories of depression (the idea that depression is caused by low levels – or “chemical imbalances” – of monoamines such as serotonin and noradrenaline) has been a centerpiece of the industry strategy to expand markets for monoamine-boosting antidepressants [38]–[41]. Importantly, however, monoamine theories, which gained popularity in the mid-1960s, have been widely criticized in light of scientific evidence [38]–[44]. We assessed whether advertisements in our sample professed monoamine theories and whether the IGM/NBL considered this a code violation. Any antidepressant advertisement claiming that depression was related to monoamine imbalances or low neurotransmitter levels was coded as professing monoamine theories.

### Swiftness of the Self-regulatory System

The rate at which the system reacted to violative antidepressant advertising was measured first by calculating the “reaction time” for each examined IGM/NBL case, defined as the time between date of original printing and date of ruling on a wrongful claim (in weeks), and second by calculating the total number of antidepressant advertisements in violation between these dates per IGM/NBL case. While the former measures the time during which companies are allowed to run misleading advertisements prior to the IGM/NBL ruling, the latter measures the number of misleading advertisements that are allowed in print. It is important to bear in mind that the reaction time represents the sum of the elapsed time between (1) the printing and reporting dates, and (2) the reporting and ruling dates. Because case reports normally do not specify reporting dates, we could not evaluate (1) or (2); however, according to the IGM, in most cases the time between reporting and ruling is less than a month [45]. To investigate if any shifts in the system’s rate of response had occurred over the ten-year period, reaction times and the number of advertisements per IGM/NBL case were plotted against date of ruling. We also investigated differences in these variables between, on one side, cases initiated via active monitoring of promotional material by the IGM and, on the other side, cases initiated after voluntary complaints from industry, health professionals, or the MPA. Additionally, we assessed whether drug companies complied with the IGM/NBL decision by determining, for each examined IGM/NBL case, the elapsed time between the last date of allowed dissemination of a claim, as specified by the IGM/NBL case report, and the last date of printing of a claim, as well as the number of advertisements printed between these dates.

### Cost Calculations

The economic cost of publishing violative antidepressant advertisements was calculated by considering the magnitude of the administrative fines in relation to antidepressant sales in Sweden. Data on administrative fines were obtained from IGM/NBL case reports [32]. Data on antidepressant sales prior to 1999 were acquired from [46] and after 1999 from [47].

Another way of gauging the economic cost of publishing misleading advertisements is to consider the magnitude of the administrative fines in relation to the cost of buying an advertisement. To this end, we estimated the additional average cost added by administrative fines to each purchased violative antidepressant advertisement. In a first step, we estimated the cost of antidepressant advertising using advertising rates published in the Swedish Medical Journal. In 1997, for example, a regular one-page color advertisement cost roughly €3100. We were unable to establish advertising rates for every year, and when queried, the journal failed to provide us with the missing data. For missing years, we estimated rates based on overall yearly rate increments. To calculate the cost of violative antidepressant advertising, we used these rate estimates together with the coded information on the rate category of each violative advertisement (see above). In a second step, we divided total administrative fines by the total cost of violative antidepressant advertising.

Finally, we investigated whether any shifts in additional average costs had occurred over the ten-year period. For IGM/NBL cases involving advertisements printed over numerous years, administrative fines were allocated between years according to the proportion of advertisements printed each year, e.g. if equal number of advertisements were printed in 2000 and 2001, the fine was divided equally between years. In all calculations, exchange rates available from the Swedish Riksbank [48] were used to convert from Swedish kronor to euro (EUR) on an annual basis.

### Statistics

Shifts in variables over the ten-year period were tested using linear regression. Differences between groups were analyzed by non-parametric Mann–Whitney test. Statistical analyses were performed using GraphPad Prism 5.0 (Graphpad software, La Jolla, CA, USA).

## Results

### Misleading Antidepressants Advertising in the Swedish Medical Journal

**Figure 2:**
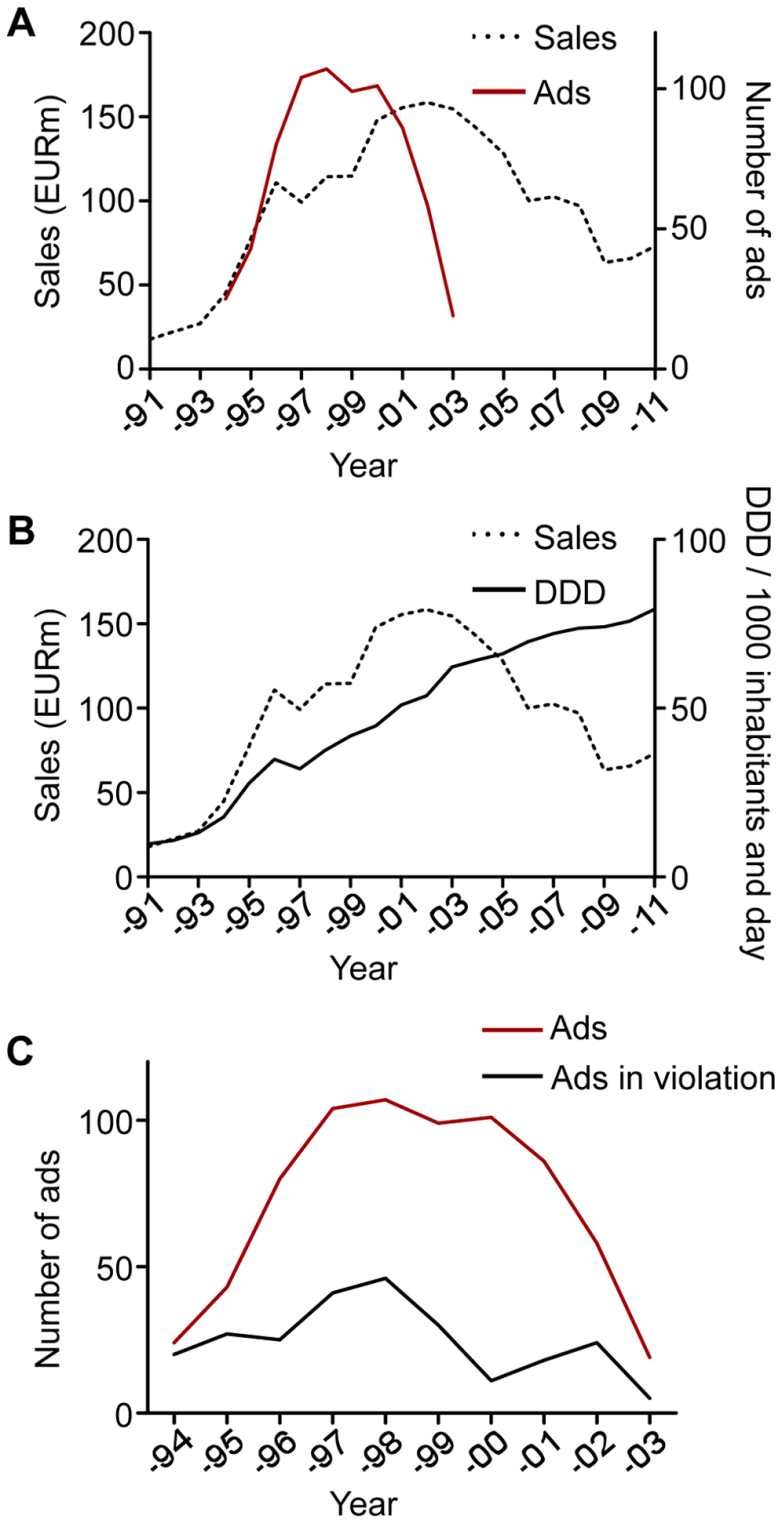
Antidepressant sales in Sweden and advertising dynamics in the Swedish Medical Journal. (A) Graphs show antidepressant sales (in EUR million) (dotted line) in Sweden in 1991–2011 and total antidepressant advertisements (red unbroken line) in the Swedish Medical Journal in 1994–2003. Spending on antidepressants rose steeply between 1993 and 1996, as well as between 1999 and 2001, but subsequently plateaued and started falling, coinciding with a drop in antidepressant advertising. (B) Declining antidepressant spending (dotted line) and advertising did not coincide with a drop in prescriptions (unbroken line), but instead coincided with price cuts due to major patent expiries. (C) Graphs show total (red line) and violative (black line) antidepressant advertising in the Swedish Medical Journal in 1994–2003.

Antidepressant sales in Sweden increased 3.4 fold between 1994 and 2003, reaching €158 million in 2002, but subsequently started falling (Fig. 2A). Over the same period, a total of 722 advertisements for antidepressants were published in the Swedish Medical Journal, corresponding to 124 unique advertisements for 13 different products (Table 1). The number of advertisements peaked in 1998 when 107 advertisements were published (Fig. 2A). Declining antidepressant spending and advertising were not associated with fewer prescriptions for antidepressants (Fig. 2B), but instead coincided with price cuts due to major patent expiries, including patents for Fontex (fluoxetine), Cipramil (citalopram) and Seroxat (paroxetine) [29].

**Table 1:** Number of printed and violative antidepressant advertisements in the Swedish Medical Journal 1994–2003.

		Antidepressant ads		Ads in violation of code	
Product	Company	Unique ads (n = 124)	Total ads(n = 722)	Unique ads(n = 47)	Total ads (n = 247)
Citalopram (Cipramil)	Lundbeck	25 (20.2%)	171 (23.7%)	12 (25.5%)	88 (35.6%)
Escitalopram (Cipralex)	Lundbeck	10 (8.1%)	42 (5.8%)	5 (10.6%)	21 (8.5%)
Fluoxetine (Fontex)	Eli Lilly	16 (12.9%)	71 (9.8%)	6 (12.8%)	8 (3.2%)
Fluoxetine (Fluoxetine Selena)	Selena	1 (0.8%)	14 (1.9%)	0	0
Fluoxetine (Seroscand Fluoxetine)	Seroscand	2 (1.6%)	15 (2.1%)	0	0
Fluvoxamine (Fevarin)	Meda/Solvay	2 (1.6%)	3 (0.4%)	1 (2.1%)	1 (0.4%)
Mirtazapine (Remeron)	Organon	10 (8.1%)	70 (9.7%)	3 (6.4%)	44 (17.8%)
Moclobemide (Aurorix)	Roche	4 (3.2%)	21 (2.9%)	0	0
Nefazodon (Nefadar)	Bristol-Myers Squibb	1 (0.8%)	2 (0.3%)	0	0
Paroxetine (Seroxat)	Novo Nordisk/SmithKleinB	25 (20.2%)	73 (10.1%)	10 (21.3%)	21 (8.5%)
Reboxetine (Edronax)	Pharmacia & Upjohn	1 (0.8%)	5 (0.7%)	0	0
Sertraline (Zoloft)	Pfizer	10 (8.1%)	149 (20.6%)	2 (4.3%)	28 (11.3%)
Venlafaxine (Effexor)	Wyeth	17 (13.7%)	86 (11.9%)	8 (17.0%)	36 (14.6%)

During this ten-year period, the IGM processed 549 complaints, 22 (or 4%) of which were relevant to antidepressant advertisements in the Swedish Medical Journal. The IGM initiated 12 of these, competing companies seven, and health professionals three (all complaints jointly submitted by the same two health professionals) (Fig. 1B). The IGM ruled against the accused company in all but one case. Similarly, between 1994 and 2003, the NBL received 258 complaints, nine (3%) of which applied to antidepressant advertisements in the journal. The MPA initiated two complaints, one of which was found to breach the industry code. The remaining seven were appeals of IGM decisions. The NBL overruled one IGM decision on one occasion, to the disadvantage of the offending company. Thus, when considered together, the IGM and NBL processed 24 cases relevant to antidepressant advertisements in the journal, 23 (96%) of which were found to be in violation (see Table S1 for listing of cases).

From the above overview, it is tempting to conclude that 23 of 124 (19%) unique antidepressant advertisements breached the code according to IGM/NBL standards. This, however, would be a gross underestimate because several rulings apply to more than one unique advertisement (since in many instances the same claim can be found in more than one unique advertisement) (Table S1). When all advertisements directly named in the rulings and other advertisements for the same product and that included the same violative materials are considered, a total of 47 rather than 23 unique advertisements breached the code, reflecting more than one third (38%) of all unique advertisements (Table 1). Calculated based on total number of advertisements, this translates to 247 of 722 (34%) antidepressant advertisements. The number of violative antidepressant advertisements peaked in 1998 when 46 violative advertisements were printed, corresponding to 43% of all antidepressant advertisements that year (minimum 11% in 2000; maximum 83% in 1994) (Fig. 2C).

### Breaches Found by the IGM/NBL

Table 2 summarizes the article violations uncovered by the IGM/NBL (see Table S2 for not violated articles relevant to medical journal advertising). Notably, the total number of article breaches (n = 402) is much larger than the total number of violative advertisements (n = 247). This is because most advertisements violated more than one article (Table S1). Remarkably, 40 of 124 (32%) unique advertisements, or 201 of 722 (28%) total advertisements, breached article 4, which mandates that medicines information must be truthful and not intended to mislead.

**Table 2:** Frequency and examples of code breaches found by the IGM or NBL in antidepressant advertisements in the Swedish Medical Journal in 1994–2003.

Art	Type of breach	Specification (Adapted from the LIF code of ethics)	Cases^1^ (n = 23)	Unique ads^2^ (n = 124)	Total ads^2^ (n = 722)	Examples^3^
1	Objectivity	Information must include accurate, objective, meaningful and balanced particulars.	3 (13.0%^4^)	6 (4.8%^4^)	37 (5.1%^4^)	Seroxat (paroxetine) was presented as “the natural choice” (IGM B030/95).
2	Objectivity	The Summary of Product Characteristics adopted for a drug constitutes thefactual basis for information aboutthe drug.	3 (13.0%)	9 (7.3%)	24 (3.3%)	Seroxat ad referred to disorders other than depression - including Generalized Anxiety Disorder for which the drug lacked approval (NBL 542/00).
3	Objectivity	Information must conform to goodpractice and good taste.	1 (4.3%)	2 (1.6%)	3 (0.4%)	The expression “imbalanced depression patient” in a Fontex (fluoxetine) ad was found distasteful (IGM B048/95).
4	Truthfulpresentation	Drug information must be truthful andmay not contain any presentation inwords or pictures that directly orindirectly – by implication, omission,distortion, exaggeration or ambiguity – isintended to mislead.	20 (87.0%)	40 (32.3%)	201 (27.8%)	Ad professed that Cipramil (citalopram) was the “most selective serotonin uptake inhibitor”. IGM and NBL noted that this statement was clearly intended to lead the reader into wrongly believing that this had clinical relevance (NBL 422/95).
8	Documentation and references	Information as to the quality andefficacy of a drug shall be capable ofsubstantiation by means ofdocumentation…of a highscientific standard.	3 (13.0%)	6 (4.8%)	21 (2.9%)	Ad claimed that Fontex was “the most documented SSRI in the world” with a reference to “data on file”. When queried regarding that reference, the company responded that this referred to the evolving bulk of clinical literature on SSRIs (IGM B048/95).
10	Documentation and references	Information that contains quotations, numerical data, etc., taken from ascientific study or deals with acomparison between drugs that isbased on such a study, mustclearly contain information aboutrelevant sources and references to thedocumentation.	1 (4.3%)	4 (3.2%)	23 (3.2%)	An abstract cited to support the claim that Effexor (venlafaxine) was superior to fluoxetine did not contain a comparison between substances (IGM W119/98).
11	Documentation and references	Documentation must be cited in a balanced and fair way.	7 (30.4%)	14 (11.3%)	60 (8.3%)	A study cited to support the claim that Effexor was superior to paroxetine reported that although Effexor beat paroxetine, neither drug was better than placebo (IGM W119/98).
12	Comparisons	Drug information that includes comparisons between effects, active ingredients, costs of treatment, etc., must be presented in such a way that the comparison as a whole is fair.	4 (17.4%)	6 (4.8%)	32 (4.4%)	A study cited to support the claim that Cipralex (escitalopram) had an earlier onset of action than Cipramil did not support this claim (NBL 626/02).
16–20	Specific rules of conduct	E.g. drug information should cite a drug’s active ingredient, dosage form and any required warnings.	1 (4.3%)	1 (0.8%)	1 (0.1%)	Ad for Seroxal did not contain the updated catalogue text, and this was not yet available to doctors (IGM B032/95).

See Table S2 for complete list of relevant articles.

1 Refers to IGM/NBL cases. Note that some cases revealed more than one type of article breach: see Table S1.

2 A few ads had contents ruled in violation in multiple cases.

3 Case numbers in the IGM/NBL database are indicated.

4 Percentage in cell of total, e.g. 13% of IGM/NBL cases, 4.8% of unique ads, and 5.1% of total ads breached article 1.

### Evidence of Lax Oversight

The data presented above could be taken to suggest that while the Swedish regulatory system has failed to protect doctors from unreliable information on antidepressants, it has simultaneously been quite effective at identifying unreliable antidepressant advertisements. We probed the strength of the Swedish system by assessing *general claims* and *comparative claims* about antidepressant efficacy in advertisements. As summarized in Table 3, a total of 16 (13%) and 28 (23%) of 124 unique advertisements, or 136 (19%) and 154 (21%) of 722 total advertisements, fell into the first and second groups, respectively. All efficacy claims were accompanied by references to articles, posters or conference presentations. While *general efficacy claims* completely eschewed attention from the IGM/NBL, *comparative efficacy claims* were found to breach the code on numerous occasions (i.e. 54% of unique *comparative efficacy claims* were found to breach the code), which may indicate that the regulatory system is generally more alert to claims that imply a comparison between products.

**Table 3:** Statements in antidepressant advertisements advancing general or comparative efficacy in depression treatment, or professing monoamine theories.

Category	Examples: translated quotes	Unique ads (n = 124)	Total ads (n = 722)
*General efficacy claims*		16/0^1^ (12.9%^2^/0%^3^)	136/0^1^ (18.8%^2^/0%^3^)
	After only two weeks, 75% had a satisfactory effect. In the rest the dose was increased to 2×75 mg/day. Irrespective of dose 82% of all patients were classified as responders on the HAM-D scale after six weeks (Effexor (venlafaxine) ad).		
	It is evident from the literature that 60–85% of patients with major depression respond to antidepressant therapy. When treatment fails, it is often considered to be due to too low a dosage (Fontex (fluoxetine) ad).		
	Two large Swedish studies show that: 9 of 10 people with depression are cured with Zoloft [sertraline]; 8 of 10 are cured with Zoloft. (Cured ≥50% reduction of points in the MADRS-scale).		
***Comparative efficacy claims***		28/15 (22.6%/53.6%)	154/107 (21.3%/69.5%)
	In another, recently presented, placebo-controlled trail citalopram was compared with sertraline. The response to treatment was significantly better in the Cipramil group than in the sertraline group at one or more time points according to HAMD, MADRS and CGI scales.		
	Effexor XR gives 30% more symptom-free patients compared with SSRI (Fluoxetine, Paroxetine and Fluvoxamine) (10% higher remission rates compared with SSRI).		
	The corresponding figures for all patients included in the study was 76% for the sertaline group and 81% for the Cipramil patients (Cipramil [citalopram] ad).		
	*Significantly more patents are restored with Cipralex* [escitalopram] *than with Cipramil…Sustained remission is achieved a week earlier with Cipralex that with Effexor XR (IGM W502/03).* 4		
	*Already within a week the difference was significant compared with paroxetine, within two weeks with citalopram and within three weeks with* fluoxetine *(Remeron (mirtazapine) ad) (IGM W448/02).* 4		
***Monoamine theories***		12/0 (9.7%/0%)	62/0 (8.6%/0%)
	Cipramil normalizes (with its high 5HT selectivity) perturbations in the serotonergic system without simultaneously influencing other neurotransmitter systems.		
	Which of your patients suffer from serotonin deficiency and which from noradrenalin [deficiency]? That question is difficult to answer before you see the effect of the antidepressant medication that is administrated (Effexor ad).		
	Aurorix [moclobemide] restores the balance of the neurotransmitters serotonin and noradrenaline.		

1 Number of ads containing claims or theories/number of ads found in breach by the IGM/NBL for this, e.g. 16 unique ads professed general efficacy claims; zero were found to be in violation for this.

2 Percentage of ads containing claims or theories of total, e.g. 12.9% of 124 unique advertisements professed general efficacy claims.

3 Percentage of ads found in breach by the IGM/NBL of ads containing claims or theories, e.g. 16 unique ads professed general efficacy claims; zero percent were found to be in violation for this.

4 Italicized statements were found to breach the code by the IGM/NBL. Case number in the IGM/NBL database is indicated.

Notably, the IGM/NBL repeatedly allowed claims about response rates of 70–90% to go unnoticed. In fact, all *general efficacy claims* were of 75% or more. A single unique advertisement containing *comparative efficacy claims* reported response rates: 76% and 81% for sertraline and citalopram, respectively. While individual studies may support such claims, they are not supported by the data submitted by companies to the Swedish MPA for marketing authorization [33]. Basing efficacy claims on individual studies, rather than the overall bulk of available data, must be considered a code violation, especially of articles 8 and 11 (see Table 2), illustrating lax oversight by the IGM/NBL.

### Oversight of Advertisements Professing Monoamine Theories

Monoamine theories have been criticized as inconsistent with the scientific evidence [38]–[44]. We identified 12 of 124 (10%) unique advertisements, or 62 of 722 (9%) total advertisements, that professed monoamine theories (Table 3). In no case did the IGM/NBL consider the dissemination of monoamine theories to be in violation of the code.

### Swiftness of the Self-regulatory System

**Figure 3:**
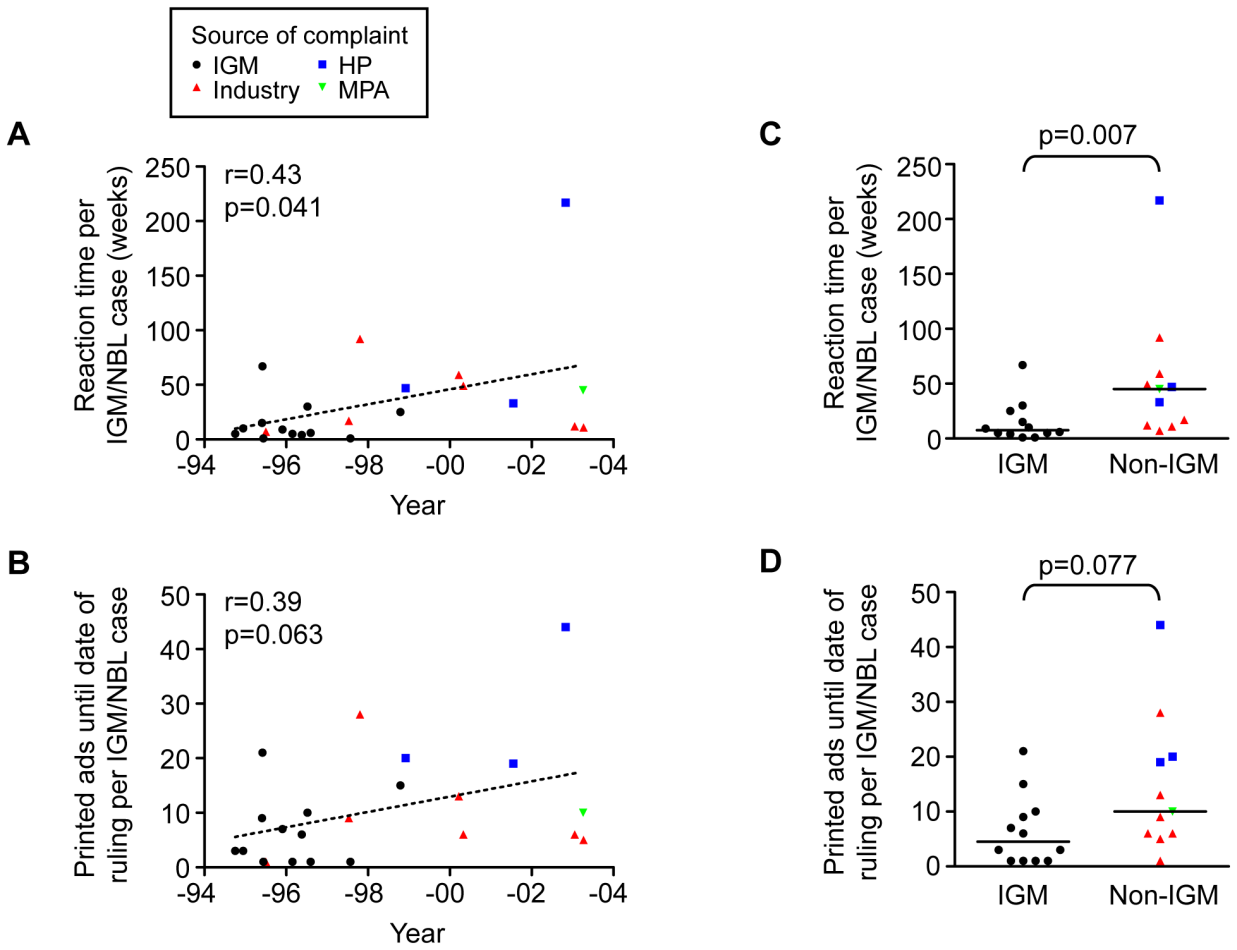
Lags in the system allowed for extended and substantial exposure to unethical antidepressant advertising. The instigator of each IGM/NBL case is indicated; HP (health professionals), MPA (Medical Products Agency). (A) Scatter plot of reaction times (i.e. the elapsed time between original publication date and date of ruling against wrongful claims) among IGM/NBL cases concerning antidepressant advertising in the Swedish Medical Journal in 1994–2003. Linear regression analysis shows that reaction times increased over the ten-year period (p = 0.041; β = 6.9 weeks/year; n = 23). (B) Scatter plot of the total number of violative advertisements in the Swedish Medical Journal per IGM/NBL case prior to date of ruling. Linear regression analysis shows that there was a borderline significant increase in the number of advertisements allowed in print over the period (p = 0.063; β = 1.4 ads/year; n = 23). (C) Reaction times and (D) number of violative advertisements among cases instigated by the IGM (i.e. via active monitoring of promotional material) and non-IGM (i.e. following voluntary complaints from industry, HP or the MPA), respectively. The median of each group is indicated by the bar. Differences between groups were analyzed with two-tailed Mann-Whitney test. There was a significant difference in reaction time between IGM and non-IGM cases (p = 0.007) (in C).

Strikingly, we found considerable variation in reaction times, i.e. the time between printing and ruling on a wrongful claim (Fig. 3A). Specifically, while median reaction time was 15 weeks, in 25% of cases reaction time was 47 weeks or more. Similarly, while the median number of printed advertisements per IGM/NBL case was seven, in 25% of cases 15 or more advertisements had been allowed in print prior to the IGM/NBL ruling (Fig. 3B). There was a small but significant trend towards longer reaction times over the studied period (p = 0.04). This trend may be related to the high proportion of cases initiated via active monitoring of promotional material by the IGM in the first half of the period and, conversely, the high proportion of cases initiated after voluntary complaints from industry, health professionals or the MPA in the second half of the period. Consistent with this, cases initiated by the IGM had shorter reaction times than cases initiated following voluntary complaints (p = 0.007) (Fig. 3C). For number of printed advertisements per IGM/NBL case between printing and ruling dates, similar but non-significant trends were observed (Fig. 3B and 3D). In sum, the evidence in this study indicates that while self-regulatory bodies may act promptly to investigate possible breaches, the system is nevertheless hampered by significant lags that allow firms to continue running violative advertisements for extended periods of time.

### Decisions Ignored and Repeat Violations

We found that, as requested by the IGM/NBL, drug companies often stopped disseminating violative antidepressant advertisements within 2–3 weeks of the first ruling. However, in three of the 23 cases, the companies ignored the IGM/NBL request (Table S1). In these cases, companies continued running advertisements for nine, 13 and 88 weeks, respectively, during which they printed five, four and 11 advertisements, respectively. This happened without additional reprimands from the IGM/NBL, lending further evidence of lax oversight. The most notable case (88 weeks; 11 advertisements) involved advertisements for Cipramil (citalopram) professing that the drug was the “most selective serotonin uptake inhibitor”; a statement clearly intended to lead the reader into wrongly believing that this had clinical relevance, both the IGM and NBL noted.

In regards to repeat violations – i.e. similar violative claims for the same drug in more than one case report – we found a single example of this (Table S1). In that case, the IGM ruled against the company twice, in 2002 and then again in 2003, for professing almost identical unsubstantiated claims about the comparative efficacy of their product Cipralex (escitalopram).

### Low Fines

The IGM/NBL regularly impose economic sanctions on companies and in 1994–2003 offenders collectively paid out €108000 in administrative fines to LIF for unethical antidepressant advertisements (Table S1). Yet the high rate of code breaches identified in this study indicates that sanctions failed to deter companies from publishing misleading antidepressant advertisements. One reason for this failure could be that fines are too low in relation to revenues generated by such misleading drug promotion [49]. Between 1994 and 2003, antidepressant sales in Sweden generated revenue of about €1.2 billion (Fig. 2A). In other words, administrative fines for unethical antidepressant advertisements corresponded to 0.009% of total antidepressant spending. Since 1994, standard fines have been ratcheted up from €1100 to today’s differentiated rates of €4500, €10200, and €15800 for simple, normal and serious offences, respectively (higher fines may be imposed for noncompliance with a ruling). However, the number of IGM/NBL rulings against offenders has not declined in response to the increased fines (Fig. S1), indicating that thus far this tactic has had little effect on corporate behavior.

**Figure 4:**
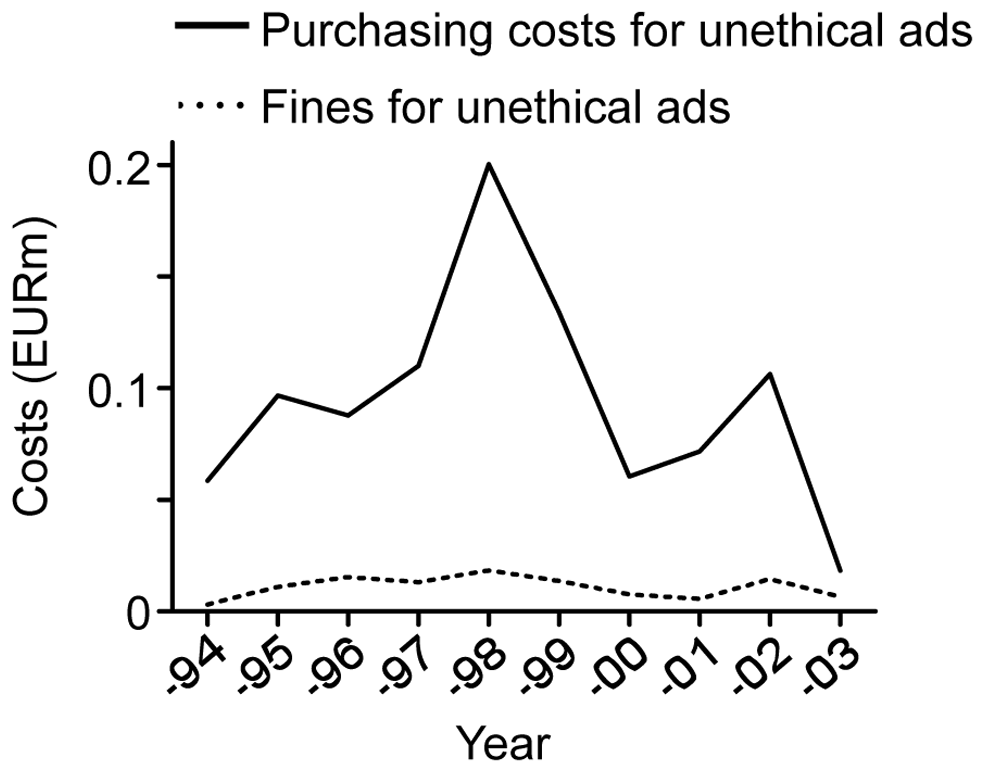
Administrative fines are low in relation to the costs of purchasing advertisements in the Swedish Medical Journal. Graphs show estimated purchasing costs for unethical antidepressant advertisements (unbroken line), according to IGM/NBL standards, in the Swedish Medical Journal in 1994–2003 and the administrative fines for offending companies (dotted line) over the same period (in EUR million).

We next considered the magnitude of the administrative fines in relation to the cost of buying an advertisement (Fig. 4). Between 1994 and 2003, companies purchased antidepressant advertisements in the Swedish Medical Journal for approximately €2.7 million, of which violative advertisements (according to IGM/NBL standards) accounted for roughly €0.94 million. This means that administrative fines added an additional average cost of 11% to each purchased violative antidepressant advertisement in the journal. There was no significant shift in the additional average cost over the studied period (p = 0.11).

## Discussion

Antidepressants comprise one of the most prescribed drug classes in the world [50]–[51]. Our analysis supports the contention that the explosion of the antidepressant market since the 1990s was associated with major promotional activity. Importantly, this study suggests that this promotional activity was to a significant extent unethical since more than every third antidepressant advertisement in our sample was unacceptable according to industry standards. Specifically, many advertisements failed to truthfully present the product. Untruthful medicines information may bias health professionals, steering them away from scientifically and economically sound prescribing practices, and thereby represent a potential danger to public health and state finances [15].

While numerous studies have addressed the low quality of medicines advertisements in medical journals, few have investigated bias in promotional regulatory regimes [9]. This study confirms prior suspicions of lax oversight on the part of self-regulatory bodies [9], [19], [23]. Thus, Swedish self-regulatory bodies, the IGM and NBL, previously heralded as exemplary [16], permitted exaggerated statements about antidepressant efficacy that, although occasionally supported by individual studies, are inconsistent with the data submitted to the Swedish MPA for marketing authorization [33]. The problem of relying on a few individual studies to substantiate claims about drug efficacy is underscored by studies showing selective publishing and selective reporting of clinical trials [34], [52]. In the past, the IGM/NBL have ruled against companies for making unjustified claims based on individual studies. Frequently, the IGM/NBL have referred to article 11 (e.g. rulings W519/03, W644/04 and W734/05), including 11.1 “the results of a study, which are contradicted by another study, may not be cited without reservation and that results that have been refuted must not be used” and 11.2: “a study may not be cited in such a way that it could convey an incorrect or misleading impression of the nature, scope, implementation or importance of the study” [26]. For example, in ruling W519/03 regarding marketing of a cyclooxygenase-2 (COX-2) inhibitor, an anti-inflammatory drug, the IGM sanctioned the offending company for disseminating material that used a single study to support a claim of superior comparative efficacy due to contradictory reports in the literature. This is pertinent to the present discussion because it suggests that the main problem is code enforcement, rather than the code itself or its interpretation.

We have also assessed oversight of adverts professing monoamine theories – a disease explanation criticized by many as incongruent with the scientific literature on depression [38]–[44], yet no IGM/NBL actions were found related to this issue. If such claims are not currently considered violations, we would argue that the IGM/NBL should revisit the evidence and consider whether these claims should be judged as misleading. The IGM/NBL have on numerous occasions ruled against companies for making claims inconsistent with the scientific evidence. Frequently, companies have been referred to articles 4 and/or 7, which mandate that drug information must be truthful and up-to-date, respectively (e.g. rulings W60/98, W448/02 and W850/06). Thus, in ruling W60/98 the IGM used the two articles to rule against a company for making claims about the protective effect of their typhoid vaccine that diverged from current evidence. Similarly to the IGM/NBL, the MPA and the European Medicines Agency have for a long time remained silent about the dissemination of monoamine theories to support the use of monoamine boosting antidepressants. However, the MPA recently responded to a letter in the Swedish Medical Journal from one of the authors of this paper, which criticized statements professing monoamine theories in Patient Information Leaflets [53], by concluding that such statements “are too categorical and simplistic, and that it would be desirable to have a more balanced description [of the etiology of depression]” [54]. Notably, as early as 2003 the Irish medicines agency prohibited drug companies from professing monoamine theories in Patient Information Leaflets [55].

This study also provides evidence of two further shortcomings that compromise the system’s ability to pressure companies into truthfully presenting their products: lags in the system and low fines. For example, regarding lags in the system, we found that in 25% of cases at least 47 weeks or more elapsed between the printing and censure of a wrongful claim. Furthermore, in three of 23 cases companies failed to comply with the obligation to stop disseminating violative material. The finding of significant lags is in accordance with the regulatory delays reported by United States Government Accountability Office (GAO) in its assessment of the effectiveness of the Food and Drug Administration (FDA) in halting the dissemination of violative direct-to-consumer advertisements [56]. Specifically, in the 19 cases from 2004 and 2005 reviewed by the GAO there was an average delay of eight months between the printing and censure of a wrongful claim. Lags in regulatory systems are clearly unacceptable since they allow plenty of time for misleading advertisements to bias prescribing practices.

Inappropriately low fines represent yet another area of major concern. Strikingly, we found that fines corresponded to as little as 0.009% of total antidepressant spending over the period, and that they added an estimated additional average cost of 11% to each purchased violative advertisement in our sample with no evidence of change over time. Arguably, such fines may merely be accepted as the “cost of doing business” and do not serve as a significant deterrent for companies with sales in the billions. Presently, fines are calculated based on the cost of administering the self-regulatory system. The fact that delinquent corporations pay administrative fines aimed at keeping the self-regulatory system afloat, rather than providing compensation for damages caused by – and profits generated as a result of – misleading drug promotion represents a major weakness in the Swedish regulatory system.

As evident from this discussion, several modifications must be made to the existing system to improve performance. For example, stricter enforcement of the code is needed, especially to ensure that professed information about drug efficacy is based on the aggregate of available data rather than on selected individual studies. Related to this, self-regulatory bodies must ensure that companies comply with their rulings. In terms of deterring companies from producing misleading information, initiating pre-vetting of promotional information by an expert body represents one possible preventive strategy; however, as the Canadian experience with pre-screening of journal advertisements by a Pharmaceutical Advertising Advisory Board demonstrates, standards need also to be sufficiently rigorous and expert bodies adequately funded to prevent misleading claims [57]. Compulsory publishing of corrective statements in the same size, placement and over the same time period as the original advertisements may also serve as a significant deterrent for companies. Most importantly, however, treating dissemination of misleading information as a criminal offence under which offending corporations pay damages rather than standard administrative fines is likely to incentivize corporate adherence to the code.

Moreover, if the system is to function efficiently, health professionals and public authorities must take greater responsibility for reporting potential breaches. Regarding public authorities, in the present case the MPA obviously failed to report inconsistencies between claims of response rates of 75–90% and the considerably more moderate effects evident from the data on which they based marketing authorization. Regarding health professionals, the current trend in Sweden is toward less reporting of breaches [58]. The failure of health professionals to report breaches in the case of antidepressant promotion is illustrated by the following example [59]. In 2000, at a national meeting of the Swedish Association of General Practice (SFAM), representing Sweden’s family doctors, an advertising campaign for Effexor (venlafaxine) won the category “worst advertisement of the year”. The campaign sought to launch Effexor as a treatment for Generalized Anxiety Disorder, but according to the SFAM the campaign was aimed at expanding markets for Effexor by medicalizing the everyday anxieties of healthy individuals. Yet no family doctor reported these advertisements to the IGM. (Incidentally, a Swedish Medical Journal jury crowned the same campaign “best advertisement of the year in the Swedish Medical Journal” because of its appeal to doctors [60]. The prize – awarded to Wyeth’s Swedish product line manager – was a weekend for two in Berlin).

Related to this, health professionals and public authorities must report potential breaches in a timely fashion. The need to improve swiftness in reporting is supported by the finding that cases initiated following voluntary complaints were associated with significant lags, possibly reflecting major gaps between dates of printing and reporting on a claim. When taken together, this points to limitations in the voluntary complaints mechanisms and, consequently, it emphasizes the need for active and efficient monitoring of promotional material by expert bodies such as the IGM to limit physician exposure to misleading medicines information.

Finally, we would suggest that measures be taken to further enhance transparency and accountability in the self-regulatory system. Specifically, we suggest that case reports not only specify all claim(s) in breach of the code, but also indicate all publications where these claims had been printed prior to the violation ruling. This would facilitate investigations of lags in the system and help to assess the impact of misleading promotion.

Admittedly, the present discussion has pointed to significant shortcomings in the Swedish self-regulatory system. It is important to keep in mind, however, that promotional regulatory regimes may vary considerably among countries and therefore caution must be exercised when generalizing results. In the United States, for example, it is the Division of Drug Marketing and Advertising within the FDA that regulates pharmaceutical advertising and promotion [8], [56]. Many countries nonetheless rely on pharmaceutical industry self-regulation [16], [18], [23]–[25]. In light of past and present praise of the efficacy and transparency of the Swedish system, there is little reason to believe that the Swedish system is any more permissive than other self-regulatory systems. However, international comparisons of the strengths and weaknesses of existing promotion regulatory systems are needed to substantiate this contention. We suggest it could be fruitful to pattern such international comparisons on this study.

Criticism of pharmaceutical industry self-regulation is far from new. In 1990, Herxheimer and Collier [17] reviwed 302 reports of complaints considered by the Association of the British Pharmaceutical Industry code of practice committee. The high frequency of serious code breaches identified, in combination with ineffective sanctions, led the authors to suggest that self-regulation mainly serves industry rather than the public, a point that resonated with the House of Commons Health Select Committee’s report on the influence of the pharmaceutical industry 15 years later [23]. Another critical appraisal of self-regulation found that, as of 1997, the Canadian self-regulatory system suffered from serious weaknesses with respect to five aspects considered important for code enforcement: (1) mechanisms for recognizing violations; (2) composition of monitoring committees; (3) sanctions for code violations; (4) the quantity and quality of information in reports issued about complaints and code violations, and (5) the circulation these reports receive [18]. The Australian self-regulatory system has come under scrutiny too, in that case for alledged problems related to retrospective detection of code breaches and low fines for violations, that is, similar to the weaknesses outlined here [61]–[62].

However, it is unclear whether such criticism has resulted in significantly stricter promotional regulatory regimes [63]. In most countries, the state holds ultimate legal responsibility to ensure that corporations adhere to existing marketing and pharmaceutical regulation, as well as to ensure safeguarding of public health. If the shortcomings uncovered by this study prove to be consistent across self-regulatory settings, and if appropriate measures are not taken to amend shortcomings, many countries might choose to reconsider the current balance between self-regulation, and direct legislative control with government oversight over medicines promotion, in an effort to protect public health and state finances. But as the reported failure of the underfunded FDA to ensure reliable pharmaceutical advertisements work to remind us, it is crucial that public regulatory bodies are adequately funded to carry out their task [8], [56]. For if adequate funding cannot be ensured, we risk substituting one problematic regulatory regime for another.

### Limitations

The main limitation of this study is its restriction to a specific drug class in a single journal. In regard to drug class, it is possible that the highly competitive nature of the antidepressant market drove companies to particularly extravagant claims. Rivalry between companies is also likely to increase motivation to complain about misleading claims (e.g. comparative efficacy claims) that are perceived to provide an advantage to competitors – but is unlikely to generate complaints about misleading claims that are mutually beneficial to all competitors (e.g. general efficacy claims or claims pertaining to monoamine theories, as addressed by this paper). Thus, future studies might appropriately address a comparison of cases with and without inter-company competition.

Concerning single journal, it should be noted that although the Swedish Medical Journal is the country’s principal medical journal, there are a few other medical journals printed in Sweden. It is reasonable to believe that advertisements in the Swedish Medical Journal at least sometimes were simultaneously printed in these journals. Consequently, the figures presented in this paper regarding the number of misleading advertisements, lags in the system and the low cost associated with misleading advertising, should be considered underestimates. This fact, however, merely serves to further strengthen the argument that pharmaceutical industry self-regulation in Sweden has not been working satisfactorily.

### Conclusions

The Swedish self-regulatory system of medicines promotion largely failed to motivate industry into providing truthful information on antidepressants in medical journal advertisements. Specifically, we demonstrate that this failure was associated with: (1) lax oversight, (2) lags in the regulatory system, and (3) low fines for violations. If current corporate regulatory regimes fail to deter industry from providing unreliable information, we suggest that many countries may want to reconsider the current balance between self-regulation, and direct legislative control with government oversight over medicines promotion, in order to ensure rational drug prescribing practices, provided that sufficient funding for public regulatory bodies is guaranteed.

## Supporting Information

Figure S1 **Violations found by the Pharmaceutical Industry Information Examiner (IGM) and the Information Practices Committee (NBL), 1994–2011.** Increased administrative fines did not result in fewer violations. Data from the Swedish Association of the Pharmaceutical Industry (LIF) database [32].
(TIF)

Table S1 **Compiled data on IGM/NBL cases.**
(PDF)

Table S2 **Frequency and examples of code breaches found by the IGM or NBL in antidepressant advertisements in the Swedish Medical Journal in 1994–2003.** Complete Table.
(PDF)
