# Supplementary figures and images for: Misleading Advertising for Antidepressants in Sweden: A Failure of Pharmaceutical Industry Self-Regulation
Source: PLoS One. 2013 May 1;8(5):e62609. doi: 10.1371/journal.pone.0062609 (PMC3641086; doi:10.1371/journal.pone.0062609)

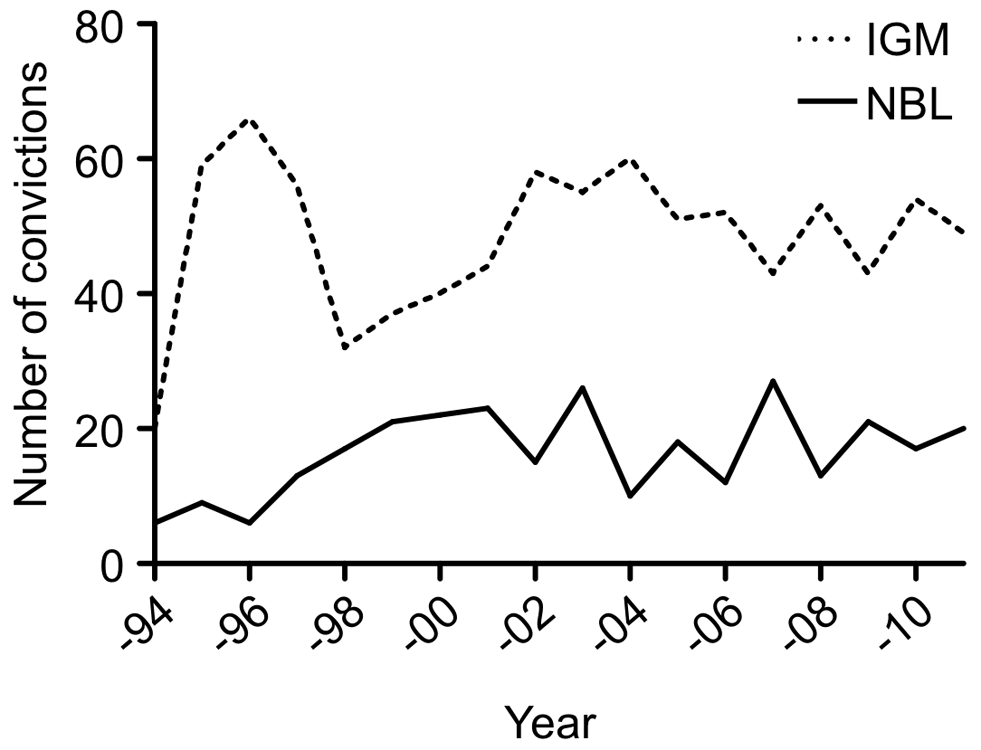

Supplement: Figure S1 — Violations found by the Pharmaceutical Industry Information Examiner (IGM) and the Information Practices Committee (NBL), 1994–2011. Increased administrative fines did not result in fewer violations. Data from the Swedish Association of the Pharmaceutical Industry (LIF) database [32]. (TIF) [file pone.0062609.s001.tif]
