# Supplementary material for: Misleading Advertising for Antidepressants in Sweden: A Failure of Pharmaceutical Industry Self-Regulation
Source: PLoS One. 2013 May 1;8(5):e62609. doi: 10.1371/journal.pone.0062609 (PMC3641086; doi:10.1371/journal.pone.0062609)
Supplement: Table S1 — Compiled data on IGM/NBL cases. (PDF) [file pone.0062609.s002.pdf]

# Table S1. Compiled data on IGM/NBL cases.

| Case no. | Product | Instigator | Sanctioned company | Ruling date | First ad[1] | Last ad[1] | Fines (SEK) | Unique ads[2] | Total ads[2] | Violated article(s)[3] |
|---|---|---|---|---|---|---|---|---|---|---|
| **IGM B005/94** | Seroxat | IGM | Novo Nordisk | 1994-10-01 | 1994; 35 | 1994; 39 | 10000 | 1 | 3 | 4 |
| **IGM B019/94** | Seroxat | IGM | Novo Nordisk | 1994-12-12 | 1994; 41 | 1994; 45 | 10000 | 1 | 3 | 4; 12 |
| **IGM B030/95** | Seroxat | Lundbeck | Novo Nordisk | 1995-07-03 | 1995; 21 | 1995; 21 | 10000 | 1 | 1 | 1; 4; 12 |
| **NBL 421/95** | | | | 1995-09-29 | | | 50000 | | | |
| **IGM B029/95[4]** | Cipramil | IGM | Lundbeck | 1995-06-04 | 1994; 8 | 1997; 10 | 10000 | 7 | 32 | 1; 4 |
| **NBL 422/95[4]** | | | | 1995-10-20 | | | 10000 | | | |
| **IGM L026/95** | Zoloft | IGM | Pfizer | 1995-05-28 | 1995; 7 | 1995; 17 | 10000 | 1 | 9 | 1; 4; 11 |
| **IGM B032/95** | Seroxat | IGM | Novo Nordisk | 1995-06-11 | 1995; 23 | 1995; 23 | 10000 | 1 | 1 | 18 |
| **IGM B048/95** | Fontex | IGM | Eli Lilly | 1995-11-27 | 1995; 40 | 1995; 49 | 10000 | 5 | 7 | 3; 4; 8; 11 |
| **IGM L004/96** | Cipramil | IGM | Lundbeck | 1996-02-23 | 1996; 4 | 1996; 4 | 25000 | 1 | 1 | 4; 8; 11 |
| **IGM B048/96** | Fevarin | IGM | Meda AB | 1996-08-07 | 1996; 26-27 | 1996; 26-27 | 15000 | 1 | 1 | 4 |
| **IGM B059/96** | Cipramil | IGM | Lundbeck | 1996-07-07 | 1995; 50 | 1996; 47 | 25000 | 3 | 10 | 4 |
| **IGM L030/96** | Effexor | IGM | Wyeth | 1996-05-17 | 1996; 17 | 1996; 23 | 25000 | 2 | 6 | 4; 11 |
| **IGM B038/97[4]** | Cipramil | Novo Nordisk | Lundbeck | 1997-07-13 | 1997; 12 | 1997; 41 | 25000 | 1 | 14 | 4; 8 |
| **NBL 460/97[4]** | | | | 1997-10-07 | | | No extra fee | | | |
| **IGM W030/97** | Cipramil | Novo Nordisk | Lundbeck | 1997-10-19 | 1996; 3 | 1997; 43 | 50000 | 4 | 28 | 12 |
| **NBL 465/97** | | | | 1997-12-18 | | | 50000 | | | |
| **IGM B041/97** | Fontex | IGM | Eli Lilly | 1997-07-27 | 1997; 30-31 | 1997; 30-31 | 25000 | 1 | 1 | 4; 11 |
| **IGM W110/98** | Cipramil | IGM | Lundbeck | 1998-10-14 | 1998; 18 | 1998; 45 | 50000 | 1 | 15 | 4; 11 |
| **NBL 496/98** | | | | 1998-12-21 | | | 50000 | | | |
| **IGM W119/98[4]** | Effexor | Health Prof. | Wyeth | 1998-11-30 | 1998; 3 | 1999; 11 | 50000 | 4 | 24 | 10; 11 |
| **IGM W221/00** | Seroxat | Wyeth | SmithKleinB | 2000-03-20 | 1999; 6 | 2000; 14 | 50000 | 6 | 13 | 2; 4 |
| **NBL 542/00** | | | | 2000-07-12 | | | 50000 | | | |
| **IGM W224/00** | Effexor | SmithKleinB | Wyeth | 2000-04-29 | 1999; 21 | 2000; 8 | 50000 | 2 | 6 | 4 |
| **IGM W293/00** | Zoloft | Health Professionals | Pfizer | Not avail. | 2000; 48 | 2001; 24 | No violation | 1 | 19 | 4 |
| **NBL 574/01** | | | | 2001-07-20 | | | 50000 | | | |
| **IGM W448/02** | Remeron | Health Prof | Organon | 2002-10-30 | 1998; 36 | 2002; 41 | 60000 | 3 | 44 | 4 |
| **IGM W478/02[5]** | Cipralex | Wyeth | Lundbeck | 2003-01-19 | 2002; 44 | 2002; 52 | 60000 | 1 | 6 | 2; 4 |
| **NBL 626/02** | Cipralex | MPA | Lundbeck | 2003-04-04 | 2002; 22 | 2002; 43 | 60000 | 2 | 10 | 4; 12 |
| **IGM W502/03[5]** | Cipralex | Wyeth | Lundbeck | 2003-04-07 | 2003; 5 | 2003; 10 | 60000 | 2 | 5 | 2; 4 |

[1] Year and issue (week) of the journal is indicated.
[2] A few advertisements had contents ruled in violation in multiple cases.
[3] For a few cases not all article violations specified in the case report applied to all advertisements encompassed by the ruling.
[4] Cases where the company ignored the IGM/NBL request to stop disseminating violative advertisements.
[5] Cases of repeat violations, i.e. similar violative claims for the same drug in more than ruling.
