# Supplementary material for: Misleading Advertising for Antidepressants in Sweden: A Failure of Pharmaceutical Industry Self-Regulation
Source: PLoS One. 2013 May 1;8(5):e62609. doi: 10.1371/journal.pone.0062609 (PMC3641086; doi:10.1371/journal.pone.0062609)
Supplement: Table S2 — Frequency and examples of code breaches found by the IGM or NBL in antidepressant advertisements in the Swedish Medical Journal in 1994–2003. Complete Table. (PDF) [file pone.0062609.s003.pdf]

Table S2. Frequency and examples of code breaches found by the IGM or NBL in antidepressant advertisements in the Swedish Medical Journal in 1994-2003. Complete Table.

Art	Type of breach	Specification (Adapted from the LIF code of ethics)	Cases[1] (n=23)	Unique ads[2] (n=124)	Total ads[2] (n=722)	Examples[3]
1	Objectivity	Information must include accurate, objective, meaningful and balanced particulars.	3 (13.0%[4])	6 (4.8%[4])	37 (5.1%[4])	Seroxat (paroxetine) was presented as "the natural choice" (IGM B030/95).
2	Objectivity	The Summary of Product Characteristics adopted for a drug constitutes the factual basis for information about the drug.	3 (13.0%)	9 (7.3%)	24 (3.3%)	Seroxat ad referred to disorders other than depression - including Generalized Anxiety Disorder for which the drug lacked approval (NBL 542/00).
3	Objectivity	Information must conform to good practice and good taste.	1 (4.3%)	2 (1.6%)	3 (0.4%)	The expression "imbalanced depression patient" in a Fontex (fluoxetine) ad was found distasteful (IGM B048/95).
4	Truthful presentation	Drug information must be truthful and may not contain any presentation in words or pictures that directly or indirectly – by implication, omission, distortion, exaggeration or ambiguity – is intended to mislead.	20 (87.0%)	40 (32.3%)	201 (27.8%)	Ad professed that Cipramil (citalopram) was the "most selective serotonin uptake inhibitor". IGM and NBL noted that this statement was clearly intended to lead the reader into wrongly believing that this had clinical relevance (NBL 422/95).
5	Identification	Drug information must be easy to recognize as such.	0	0	0	
6	Identification	Drug information must contain a clear statement about the year of publication	0	0	0	
7	Current knowledge	Drug information must be up-to-date.	0	0	0	
8	Documentation and references	Information as to the quality and efficacy of a drug shall be capable of substantiation by means of documentation…of a high scientific standard.	3 (13.0%)	6 (4.8%)	21 (2.9%)	Ad claimed that Fontex was "the most documented SSRI in the world" with a reference to "data on file". When queried regarding that reference, the company responded that this referred to the evolving bulk of clinical literature on SSRIs (IGM B048/95).
9	Documentation and references	Documentation that has been compiled for a particular drug may be cited in support of information about another drug only if the documentation is obviously applicable to the latter drug as well.	0	0	0	
10	Documentation and references	Information that contains quotations, numerical data, etc., taken from a scientific study or deals with a comparison between drugs that is based on such a study, must clearly contain information about relevant sources and references to the documentation.	1 (4.3%)	4 (3.2%)	23 (3.2%)	An abstract cited to support the claim that Effexor (venlafaxine) was superior to fluoxetine did not contain a comparison between substances (IGM W119/98).
11	Documentation and references	Documentation must be cited in a balanced and fair way.	7 (30.4%)	14 (11.3%)	60 (8.3%)	A study cited to support the claim that Effexor was superior to paroxetine reported that although Effexor beat paroxetine, neither drug was better than placebo (IGM W119/98).
12	Comparisons	Drug information that includes comparisons between effects, active ingredients, costs of treatment, etc., must be presented in such a way that the comparison as a whole is fair.	4 (17.4%)	6 (4.8%)	32 (4.4%)	A study cited to support the claim that Cipralex (escitalopram) had an earlier onset of action than Cipramil did not support this claim (NBL 626/02).
13	Discreditation	Information may not contain presentations likely to bring another drug/company into contempt or lay it open to ridicule.	0	0	0	
14-15	General rules of conduct	Drug information shall be distributed selectively.	0	0	0	

Table S2. Continued.

16-20	Specific rules of conduct	E.g. drug information should cite a drug's active ingredient, dosage form and any required warnings.	1 (4.3%)	1 (0.8%)	1 (0.1%)	Ad for Seroxal did not contain the updated catalogue text, and this was not yet available to doctors (IGM B032/95).

[1] Refers to IGM/NBL cases. Note that some cases revealed more than one type of article breach: see Table S1.
[2] A few ads had contents ruled in violation in multiple cases.
[3] Case numbers in the IGM/NBL database are indicated.
[4] Percentage in cell of total, e.g. 13% of IGM/NBL cases, 4.8% of unique ads, and 5.1% of total ads breached article 1.
